# Did group II intron proliferation in an endosymbiont-bearing archaeon create eukaryotes?

**DOI:** 10.1186/1745-6150-1-36

**Published:** 2006-12-07

**Authors:** Anthony M Poole

**Affiliations:** 1 Department of Molecular Biology & Functional Genomics, Stockholm University, SE-106 91 Stockholm, Sweden

## Abstract

Martin & Koonin recently proposed that the eukaryote nucleus evolved as a quality control mechanism to prevent ribosome readthrough into introns. In their scenario, the bacterial ancestor of mitochondria was resident in an archaeal cell, and group II introns (carried by the fledgling mitochondrion) inserted into coding regions in the archaeal host genome. They suggest that if transcription and translation were coupled, and because splicing is expected to have been slower than translation, the effect of insertion would have been ribosome readthrough into introns, resulting in production of aberrant proteins. The emergence of the nuclear compartment would thus have served to separate transcription and splicing from translation, thereby alleviating this problem. In this article, I argue that Martin & Koonin's model is not compatible with current knowledge. The model requires that group II introns would spread aggressively through an archaeal genome. It is well known that selfish elements can spread through an outbreeding sexual population despite a substantial fitness cost to the host. The same is not true for asexual lineages however, where both theory and observation argue that such elements will be under pressure to reduce proliferation, and may be lost completely. The recent introduction of group II introns into archaea by horizontal transfer provides a natural test case with which to evaluate Martin & Koonin's model. The distribution and behaviour of these introns fits prior theoretical expectations, not the scenario of aggressive proliferation advocated by Martin & Koonin. I therefore conclude that the mitochondrial seed hypothesis for the origin of eukaryote introns, on which their model is based, better explains the early expansion of introns in eukaryotes. The mitochondrial seed hypothesis has the capacity to separate the origin of eukaryotes from the origin of introns, leaving open the possibility that the cell that engulfed the ancestor of mitochondria was a sexually outcrossing eukaryote cell.

## Background

In two papers published this year (one on Biology Direct [1], and one coauthored by W. Martin in Nature [2]), Eugene Koonin argues that intron proliferation played a key role in the evolution of eukaryotes from an ancestral archaeon. I argue here that an archaeal host is not compatible with intron proliferation under the introns late model for the origin of introns.

In brief, Koonin's model proposes the following steps in the evolution of modern eukaryotes:

1. Endosymbiosis: a bacterium of α-proteobacterial origin takes up residence in an archaeal host, by an unspecified mechanism.

2. Endosymbiont to host gene transfer: group II self-splicing introns resident in the endosymbiont are transferred to the genome of the archaeal host, and subsequently proliferate in the host genome.

3. Intron proliferation results in selection for improved mechanisms of quality control – compartmental separation of splicing and translation via evolution of the nuclear envelope, nonsense-mediated decay, ubiquitin-dependent protein degradation – all of which serve to reduce formation of aberrant polypeptides via translational readthrough of intron-disrupted open reading frames.

The series of events described by Koonin in point 3 build on points 1 and 2, namely, that the host cell was an archaeon, and that group II introns entered the archaeal host genome, inserted into open reading frames and proliferated greatly in number. These two points are not interdependent, and a criticism of one does not therefore invalidate the other.

Here I raise two criticisms of Koonin's model. The first concerns point one above. I argue that current observations of contemporary and past endosymbioses do not readily support an archaeal host and a protoeukaryotic host does not conflict with the absence of extant archezoa. Point two relates to the likelihood of group II intron spread in an archaeon, where I point out that both theory and observation speak against proliferation and insertion into open reading frames in an asexual host. In advocating the first two points, Koonin [1] and Martin & Koonin [2] inadvertently eliminate the strongest support for the introns late hypothesis. Aspects of Koonin's model (point 3 above) may still hold, but both theory and data indicate that massive proliferation of selfish elements only occurs in lineages with meiotic sex. My conclusion is that large scale intron proliferation would only be possible in a sexual 'protoeukaryote'.

It is worth noting at this point that a model bearing similarities to the Koonin/Martin model has been published independently by López-García & Moreira [3]. While I focus this critique on the Koonin/Martin model, some of the issues raised are likewise relevant to aspects of the López-García/Moreira model.

## Could group II introns proliferate aggressively in an archaeon?

Koonin suggests that group II introns entered an archaeal host via the α-proteobacterial endosymbiont. The consequence is an explosion in intron numbers, compensatory evolution of a number of mechanisms of transcript quality control (the nucleus [2], nonsense-mediated decay (NMD), ubiquitinylation), and side-effects such as the evolution of linear chromosomes with telomeres and telomerase.

The important point here is that group II introns, upon arrival in the archaeal host have, 'apparently, gone berserk within the host cell' (see also [2]). Like the mitochondrial seed hypothesis [4,5], upon which Koonin's model is based, the argument is based on two premises. First, that group II introns are the ancestors of spliceosomal introns and the spliceosome, and second, that introns would proliferate in the genome of the host (under both models this can apply both to group II intron spread and later spliceosomal intron spread after the evolution of the spliceosome). Regarding the first point, there is plenty of circumstantial evidence that can be interpreted in favour of a common ancestry. Alternative interpretations are nevertheless possible, and I discuss this briefly in the referee report that accompanies Koonin [1]. I will not reiterate that point here; the current discussion is best served by assuming that group II introns did indeed enter the eukaryote lineage via the mitochondrial ancestor, later evolving into the spliceosome and spliceosomal introns.

The second premise is that group II introns would have proliferated in the host genome. Both papers [1,2] argue that introns would have been selectively disadvantageous; below I describe why this is important, but here is the reasoning given for the selective disadvantage of intron insertion. If host transcription and translation are coupled, and excision of newly inserted introns is the slowest step in production of a functional message, the ribosome runs the risk of reading through into the intron before it has been spliced out. This can therefore result in formation of aberrant proteins.

A further key feature of this model is that the source of group II introns invading any host genome is the mitochondrion; spread of group II introns between individuals (i.e. intergenomic spread) is not invoked. The model permits any host genome within the population to receive group II introns from their mitochondria (endosymbiont gene transfer), so transfer is iterative and ongoing. However, the main source of intron proliferation is spread of introns already integrated into the host genome (intragenomic spread). According to the model, the small effective population size of this new 'prekaryotic chimera' means it is not possible to eliminate group II intron proliferation by purifying selection; drift will dominate, and individuals in which intron proliferation is occurring will be fixed, in spite of their reduced fitness.

That genetic elements can proliferate at the expense of the fitness of the host in which they are found is now widely accepted. However, this is only predicted to occur under certain circumstances. A model published by Hickey [6], illustrates under which circumstances this will occur, and furthermore shows that spread is not predicted under the conditions invoked in the Koonin/Martin model. Moreover, a documented case of recent group II intron invasion into archaea fits with Hickey's model, not the Koonin/Martin model. Finally, I will point out that the mitochondrial seed hypothesis (upon which the Koonin/Martin model is based, but which in contrast allows the host to be a sexual eukaryote) is compatible with the predicted behaviour of selfish genetic elements described by Hickey.

What follows is a brief summary of the relevant aspects of Hickey's model, which I will relate back to the Koonin/Martin model. The first point concerns the nature of intragenomic spread. Hickey defines the average copy number per cell (*f*) of a transposable element in a population as:

f=abN
 MathType@MTEF@5@5@+=feaafiart1ev1aaatCvAUfKttLearuWrP9MDH5MBPbIqV92AaeXatLxBI9gBaebbnrfifHhDYfgasaacH8akY=wiFfYdH8Gipec8Eeeu0xXdbba9frFj0=OqFfea0dXdd9vqai=hGuQ8kuc9pgc9s8qqaq=dirpe0xb9q8qiLsFr0=vr0=vr0dc8meaabaqaciaacaGaaeqabaqabeGadaaakeaacqWGMbGzcqGH9aqpdaWcaaqaaiabdggaHjabdkgaIbqaaiabd6eaobaaaaa@32D4@

where *a *is the average copy number of the element per genome, counting only those genomes with at least one copy; *b *is the number of genomes containing one or more copies; *N *is the population size (number of genomes).

*f *can increase via an increase in either *a *or *b*. If *f *increases due to an increase in *a*, this is intragenomic spread; the element is increasing in frequency only because those genomes containing copies now contain even higher numbers of the element. In this case, which describes an asexual population, elements do not spread to new genomes. Consequently, those individuals with harmful elements (as per Koonin/Martin) will be at a selective disadvantage relative to those without, and element-carrying individuals are not predicted to spread within the population.

The parameter *b *is not irrelevant to the Koonin/Martin model. While the model does not include intergenomic spread between individuals, endosymbiont gene transfer of group II introns enables *b *to increase in the absence of intergenomic spread. Therefore, it is possible for group II introns to be fixed in the short term, by drift or by high rates of endosymbiont gene transfer.

This is unsurprising in that we know that group II introns exist in both bacterial and archaeal lineages, and are able to spread via horizontal gene transfer. However, what is not observed is massive proliferation within these asexual lineages, even in the presence of horizontal gene transfer.

A case in point is the recent discovery of group II introns in two species of archaea, *Methanosarcina acetovorans *and *Methanosarcina mazei *[7,8]; in both cases, group II introns have become established as the result of horizontal gene transfer from bacteria. This provides an ideal analogue to the 'primitive prekaryote' host genome, a garden-variety archaeon. Assuming rates of gene transfer and proliferation are similar to *Methanosarcina *spp. and that these species are sufficiently 'garden-variety', one would expect a similar rate of proliferation as in the Koonin/Martin model. However, none of the group II introns are inserted in archaeal open-reading frames, hence do not result in ribosome readthrough. Instead, these genes have a tendency to insert into the reverse transcriptase genes encoded by other group II introns, generating nested introns. I have no idea as to the effective population size of these two archaea, but that these elements have neither gone berserk (4 in *M. mazei*, 21 in *M. acetovorans*), nor inserted into archaeal protein-coding genes does not serve to strengthen the model presented by Koonin.

Rather, this is what one may expect for selfish elements in asexual lineages, even with horizontal gene transfer. Upon insertion, such elements will end up in linkage disequilibrium with the other genes in the genome (there is no meiotic recombination and no outbreeding). Consequently, in the long term, even if drift fixes the presence of an element that imparts a cost to the host, elements that evolve to be less harmful will be at a selective advantage; the fitness of the host and the element are identical. Thus, rather than spreading wildly, the result will be more cautious mechanisms of maintenance or spread. That the group II introns from *Methanosarcina *spp. have not inserted into coding regions is probably consistent with this. Likewise, assuming some cost associated with element presence, individuals that completely lose the elements will be at an advantage [6,9].

If an element does proliferate wildly in an asexual population, the cost to all individuals may become so high as to lead to population extinction. Indeed, it has recently been argued that element overload is one probable cause of extinction of obligately asexual lineages that have evolved from sexual lineages [10]. Survival would entail loss of those elements with the highest cost (i.e. that proliferate greatly in number). Consistent with this is the observation that retrotransposons appear to have been completely lost from Bdelloid rotifers, which evolved from sexual ancestors some 80 million years ago [11]. The same picture is seen for *Giardia lamblia*, which is not known to be sexual [12], and which, incidentally, is very intron-poor, with only three introns identified to date [13,14].

The bottom line is that even if endosymbiont transfer can ensure an increase in the proportion of individuals carrying one or more genomic copies of an element, there will still be a selective advantage for attenuation, even if complete loss does not occur.

Assuming introns-late, these elements have must have proliferated at some stage during early eukaryote evolution. Again, Hickey's paper explains under which circumstances this can occur, even when the element has a deleterious effect on the fitness of the host. In an attempt to avoid reproducing the entire paper here, the key point is that this will happen in a diploid, sexual, outbreeding population.

Individuals carrying the element, despite having a lower fitness relative to element-free individuals, will nevertheless dominate the population. Considering just a single locus, when the frequency of the element in the population is close to zero, the element will double in frequency within the population because most zygotes receive a single copy (they begin as heterozygotes) but pass on twice as many copies per gamete (transposition makes them homozygous for the element). If they are only half as fit as element-free individuals (i.e. they pass on only half as many gametes), the number of copies of the element that are passed on is the same for a normal nontransposable gene. Consequently, provided the cost to the host is < 0.5, and with high efficiency of transposition, the element will spread. Hickey's model illustrates that, as the element increases in the population, selection against individuals can rise significantly above 0.5, yet this will not impede further spread.

What this is telling us then is that meiotic sex and outbreeding are prerequisites for introns to have proliferated massively under introns-late. For Koonin's theory to work over a long enough time scale for several complex systems of quality control to emerge, I would argue that he should at least have invoked the emergence of facultative meiotic sex. The problem with this is that sex, with its two-fold reproductive cost, must be invoked under a scenario where there is a population of primed selfish elements 'waiting' to spread. While the level of sexuality can be increased in a facultatively sexual population, this is not so for an asexual population [9]. As the ever-present difficulty with models for the origin of sex is accounting for the short-term selective advantage for sex, this would represent a rather backwards way of approaching the problem!

## Saving introns-late

Invoking a garden-variety (asexual) archaeon as Koonin does serves to weaken the introns-late hypothesis because it eliminates the possibility of the mitochondrion entering an early eukaryote stem lineage that had already evolved meiosis (see [15] for recent discussion on the timing of the evolution of meiosis). Contrary to what Koonin [1] and Martin & Koonin [2] advocate, it is not certain that eukaryotes evolved directly from archaea, a point addressed in detail elsewhere [16], and which I summarise in three points here.

I will begin by stating two points of broad agreement. There is overwhelming phylogenetic evidence for the α-proteobacterial origin of mitochondria and related organelles, but this is not true for an archaeal origin of eukaryotes. Key evidence for α-proteobacterial origin of eukaryotes comes from phylogenetic studies which show that mitochondrial genes are most closely related α-proteobacterial genes [17]. In addition to the clear morphological similarities between mitochondria and bacteria, numerous observations such as gene order conservation and bacterial-like ribosomes serve to emphasise the similarity between mitochondria and bacteria [17].

It is likewise accepted that there are numerous similarities between archaea and eukaryotes [18] – eukaryote-like histones have been found in both euryarchaea and crenarchaea [19]; archaea and eukaryotes make use of small RNAs (known as small nucleolar or snoRNAs in eukaryotes) to guide ribose methylation and pseudouridylation of rRNA [20]; and similarities have been observed between archaeal and eukaryotic DNA replication machinery [21,22].

What is less widely discussed is that these similarities can be accounted for by several scenarios, all of which can be tested phylogenetically. The first scenario is that archaea and eukaryotes are sister groups. In other words, each is monophyletic, and the two groups share a common ancestor. The second is that eukaryotes evolved directly from an archaeal ancestor, and thirdly, archaea could have evolved directly from a eukaryotic ancestor. No data exist to support the third suggestion, and to my knowledge, no one has ever argued that archaea evolved directly from eukaryotes; I include it solely for completeness. Direct evidence for the second suggestion would be that at least some eukaryote genes with archaeal orthologues are found to group within the diversity of modern archaea in a phylogenetic tree, with bacteria as outgroup. The first scenario can account for sequence similarity between archaeal and eukaryotic sequences, with the prediction that, for those sequences with sufficient phylogenetic information, the monophyly of the two domains is recovered.

If archaea had already become a distinct domain prior to the origin of eukaryotes, eukaryote nuclear genes of archaeal origin should group specifically within the diversity of modern archaea, in exactly the same way as genes of mitochondrial origin fall within the diversity of modern bacteria, showing a specific relationship to α-proteobacteria. We see this clearly for mitochondrial-origin genes, but this has not been shown to be the case for supposedly archaeal-origin genes. That many archaeal genes are similar to eukaryotic genes supports a common origin for the two domains, but does not demonstrate that eukaryotes evolved from archaea. For this to be supported requires an explicit phylogenetic affinity between eukaryote genes and orthologous genes from a specific group of archaea, for instance methanogens [23,24]. So point one is that, if eukaryotes evolved from an endosymbiosis between a bacterium and an archaeon, we should see phylogenetic evidence for this.

Point two is that no extant intermediate forms are observed that can clarify the evolution of the numerous differences between archaea and eukaryotes. The popularity of an archaeal host for the mitochondrion emerged because the archezoa, a paraphyletic group thought to have evolved before the introduction of the mitochondrion, did not diversify prior to the endosymbiosis that gave rise to mitochondria and its derivatives (mitosomes and hydrogenosomes) – all 'archezoa' either still have, or have lost, this organelle [2]. Therefore, as has been argued by numerous authors, and is now widely accepted, we need to include mitochondria in the list of eukaryote-specific features that evolved prior to the diversification of modern eukaryotes. This list includes features such as meiosis, linear chromosomes, the spliceosome and spliceosomal introns, the nucleus and nucleolus, to name but a few prominent traits. All these eukaryote-specific features have arguably evolved in the stem leading to modern eukaryotes, and our ability to conclude these are derived and that archaea represent the ancestral state requires, ergo, that phylogenetically, eukaryotes are endosymbiont-carrying archaea.

One has to accept the necessity of stem-lineage eukaryote ancestors irrespective of the absence of extant primitively amitochondriate eukaryotes. By stem lineage I do not mean the extant eukaryotes that were formerly designated archezoa. I mean lineages of eukaryotes that have gone extinct and which diverged from the lineage leading to the Last Eukaryotic Common Ancestor (see [25] for standard definitions of stem and crown groups). This is because, even if (as Koonin argues) the ancestors of eukaryotes were archaea bearing α-proteobacterial endosymbionts, there are no intermediate stages between this hypothetical ancestor and modern eukaryotes [16].

The third point is that no archaea are known to carry endosymbionts, hence Koonin's model requires that this capacity evolved in archaea and then subsequently disappeared completely. There are no known cases modern archaea housing bacterial endosymbionts. All endosymbioses that have generated organelles (chloroplast, secondary and tertiary endosymbioses) subsequent to the mitochondrion clearly involve eukaryotes [26], and modern examples of endosymbioses involving eukaryotes are widespread. While there is one example of a bacterium within a bacterium within a eukaryote [27] this is not equivalent to a bacterial-archaeal endosymbiosis. Consequently, the entire thesis rests on the unproven ability for archaea to be capable of hosting endosymbionts.

## Concluding remarks

In summary, I am not convinced that the host in the endosymbiosis leading to modern eukaryotes was an archaeon. This should be detectable phylogenetically, picking out a specific group of archaea as the closest relatives of eukaryotes in exactly the same way as this is possible for the mitochondrion. Second, no archaea have been identified which carry endosymbiont bacteria, so accepting Koonin's assumption would require that all extant archaea have subsequently lost this capacity. Nor am I convinced that the transfer of group II introns into an archaeal host from its bacterial endosymbiont would have led to massive expansion of group II elements. This does not fit with our current knowledge of selfish element spread under an asexual reproductive mode. That modern methanogenic archaea of the genus *Methanosarcina *have not suffered from massive intron expansion seems to confirm this suspicion.

Importantly, even if eukaryotes had evolved from archaea, the model still runs counter to a considerable body of knowledge on selfish element proliferation. By invoking a sexual, eukaryotic host (as a later step in evolution from archaea), the spread of introns in eukaryotes can better be accounted for under our current understanding of selfish element spread in sexual populations. Without the conditions created by intron spread the selection pressure to drive the emergence of the eukaryotic nucleus and features for quality control must come from some other source.

## Author's note

This comment article began as a referee report I wrote when reviewing Koonin [1]. Dr. Koonin suggested that that report should instead be published as a stand-alone piece. However, this would either require that the report was withdrawn and not published alongside his paper, or that it would be duplicated in its entirety. As neither solution seemed ideal, I have rewritten the original commentary, focusing on what I view as the most pertinent points. My sincere thanks go to Dr. Koonin for fostering a climate of open and collegial debate.
